# A Case of Unresectable Rectal Necrosis

**DOI:** 10.1155/2011/212840

**Published:** 2011-05-05

**Authors:** Mohammed Nassif, Ahmed Ameer, Sarkis H. Meterissian, Ari-Nareg Meguerditchian

**Affiliations:** Department of Surgery, McGill University Health Centre, Room S10.22, 687 Pine Avenue West, Montreal, QC, Canada H3A 1A1

## Abstract

*Introduction*. Necrosis of the rectum is an uncommon finding due to abundant collateral vasculature. Its management remains challenging, without clear consensus in the literature. *Case Report*. We describe a case of a 53-year-old woman with multiple medical comorbidities that presented in septic shock and hematochezia. Colonoscopy revealed ischemic colitis. Conservative management was instituted. At two weeks, she presented evidence of peritonitis. Exploratory laparotomy revealed extensive necrosis of the left colon and rectum. Due to dense inflammation, resection was deemed unsafe. Therefore, a transverse ostomy with mucosal fistula was preformed. Multiple drains were left in place. The patient healed uneventfully. *Conclusion*. This case illustrates that, if extensive dissection of the distal colon and rectum is unsafe due to the patient's critical condition or technical feasibility, then a diverting ostomy of the proximal viable bowel along with a mucus fistula and good drainage of the abdomen represents an acceptable alternative.

## 1. Introduction

 Acute ischemic colitis is a well-described pathology and the most common form of gastrointestinal ischemia [1, 2]. It ranges from mucosal involvement only to more severe full-thickness gangrene [3]. In contrast, rectal ischemia is rare, as there are abundant collateral vessels supplying the rectum. Surgical treatment of gangrenous ischemic proctitis is controversial. The presence of dense inflammatory reaction in an area of challenging anatomy may make dissection or resection technically inadvisable.

In this paper, we present a case of severe gangrenous ischemia involving the rectum as well as the descending and sigmoid colon. That was treated without resection. We discuss and review the surgical management of severe gangrenous proctitis.

## 2. Case Report

 A 53-year-old Caucasian female was admitted to the emergency department of the McGill University Health Centre in septic shock. Her past medical history was positive for atrial fibrillation, chronic renal failure (not on dialysis), hypertension, chronic obstructive pulmonary disease, antiphospholipid syndrome on steroids, recurrent deep venous thrombosis (DVT) and pulmonary embolism (PE) with a permanent inferior vena cava filter in place, allergy to heparin, and chronic heart failure (CHF) with an ejection fraction of 30%. The patient was also known for self-mutilating behavior with recurrent injections of foreign material into a peripherally inserted central catheter and thighs, resulting in multiple recurrent admissions for sepsis. Her past surgical history was positive for a total abdominal hysterectomy, appendectomy, and inguinal hernia repair.

The patient underwent resuscitation and admission to ICU with pressure support and admitted to the ICU. Blood cultures were positive for *Burkholderia tropica*, for which she was put on broad spectrum antibiotics. Within 48 hours, she developed bright red blood per rectum. Colonoscopy revealed ischemic colitis which was managed conservatively. 

**Figure 1:**
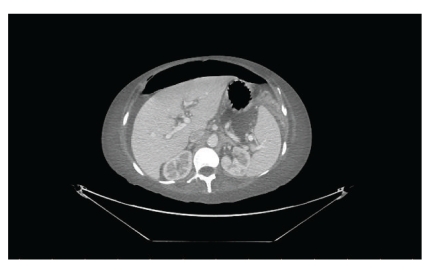
CT scan showing free air in the abdomen.

**Figure 2:**
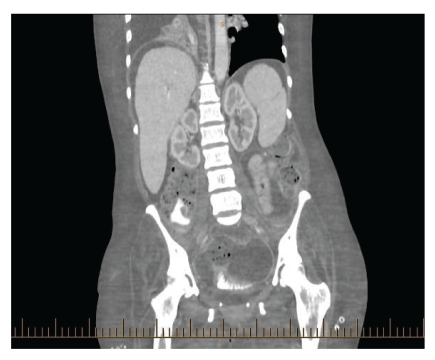
CT scan showing rectosigmoid out-pouching.

Two weeks after discharge from ICU, the patient developed sudden onset severe abdominal pain with fever and tachycardia. Abdominal examination showed generalized abdominal tenderness associated with signs of peritonitis. A computer tomography (CT) scan of the abdomen revealed free air in the abdomen (Figure 1) and an out-pouching at the level of the rectosigmoid junction (Figure 2).

After an urgent surgical consultation, the patient was taken to the operating room (OR) for an emergency exploratory laparotomy. Intraoperative findings included extensive necrosis of the descending and sigmoid colon all the way to and including the peritoneal reflection. Rectal examination also revealed necrosis of the rectum and anus with absent sphincter tone. Furthermore, dense inflammatory reaction precluded safe dissection of vessels and the ureter.

In light of this and given the patient's instability, it was felt that dissection and resection would be too risky, with possible hemorrhage and injury to the ureter. We, therefore, elected to mature an end transverse colostomy and mucous fistula leaving the gangrenous descending and sigmoid colon with rectum in place. Two large Jackson-Pratt (JP) drains were placed, one on the sigmoid and one in the pelvis.

**Figure 3:**
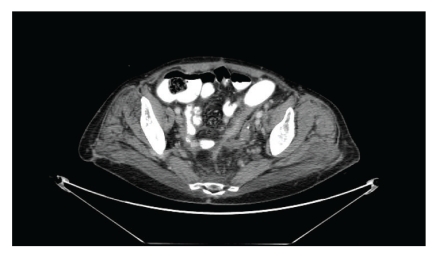
CT scan showing fibrosis of sigmoid.

After stabilization, the patient was discharged from the ICU and sent to rehabilitative services. She recovered well, apart from a small pulmonary embolism that was treated successfully with Fondaparinux. At one month after the procedure, the patient tolerated normal diet and was discharged home with a functional colostomy. A 6-month follow-up CT scan (Figure 3) revealed fibrosis of the left lower quadrant without evidence of colonic or rectal lumen.

## 3. Discussion

 Ischemic colitis (IC) is the most common form of gastrointestinal ischemia representing more than half of all cases [1, 2]. Marston et al. described different types of IC ranging from non-gangrenous IC to more severe IC with full-thickness gangrene and perforation [3]. Gangrenous IC is found in about 15% of these cases and commonly occurs at the splenic flexure and descending colon, also known as the watershed area, where limited collateral blood supply is available [3, 4]. 

The involvement of the rectum in ischemia is rare and only present in 2–5% of cases because of the presence of abundant collaterals [4, 5]. The blood supply of the rectum comes from the superior rectal artery which is a branch of the inferior mesenteric artery (IMA), middle rectal artery which is a branch of the internal iliac artery, and the inferior rectal artery which branches off the internal pudendal artery. In case of injury or ligation of the IMA, the superior mesenteric artery (SMA) maintains the blood supply to the branches of the IMA, if present [6]. Other collaterals also exist which further protect the rectum from ischemic insults. These include collaterals between superior, middle, and inferior rectal arteries, anastomoses between the internal iliac arteries, and lumbar vessels and other collaterals between the internal and external iliac arteries [7]. 

The patient presented in this case report had multiple risk factors for IC including antiphospholipid syndrome with a past history of DVT and PE, previous abdominal surgery, CHF with low EF, septic shock, and the initiation of vasopressors [8–10]. According to the literature, aortoiliac surgery (absent in this case) still remains the most common etiology of rectal ischemia due to disruption of its collateral blood supply [11].

Surgical management of gangrenous colitis is controversial, and the presence of rectal ischemia adds more to the challenge. In a report by Nelson et al. [12], four out of six patients with ischemic proctitis underwent surgery. Two patients underwent Hartmann's resection and survived with no morbidity or mortality from residual pelvic sepsis secondary to unresected residual rectum. The other two patients died, one intraoperatively after failure of conservative treatment and the other patient had delayed diagnosis and died immediately after Hartmann's resection. They concluded that severe cases of rectal ischemia or perforation require Hartmann's resection with low division of the rectum with no delayed morbidity noted in their patients after retention of the necrotic rectum. Emergency rectal resection might add additional risk to the primary procedure in an already unstable patient.

A report by Maun et al. [13] disagrees with what Nelson et al. have proposed and favors the performance of complete proctectomy in cases of rectal necrosis as retention of dead rectum may add a persistent source of sepsis. The authors describe four cases with acute ischemic proctitis. All four patients had similar presentations, a past medical history of atherosclerotic disease, and cardiac risk factors. Two out of four patients died. The first patient underwent abdomino-perineal resection (APR) as the initial procedure and died twenty seven days postoperatively because of respiratory complications. The second patient had a gangrenous rectum and underwent rectosigmoid resection with end colostomy and closure of the residual rectum. The patient did not show signs of improvement and, the abdomen was re-explored where the residual rectum was found to be necrotic and it was resected. However, the patient died after the procedure. The entire rectum was found to be dead in the other two patients who survived and both had proctectomy with endcolostomy, and the necrotic rectum was left in place.

In another series by Sharif and Hyser [14], fifteen cases of ischemic proctitis were reviewed. Eight out of the fifteen patients required surgical intervention. Five patients underwent Hartmann's procedure, one patient had proctocolectomy and transverse colostomy, and two patients underwent total abdominal colectomy and ileostomy. APR was not performed in any of the patients. The mortality for surgically treated patients was 25%, directly related to the extent of bowel resection. The authors concluded that the extent of resection is related to bowel appearance during surgery and the remaining rectal stump will slough or drain out from the perineum and agreed with Nelson et al. that APR is unnecessary during emergency and in high-risk groups.

## 4. Conclusion

Surgical treatment of patients with rectal necrosis is controversial and challenging. Rectal resection depends on many factors, starting with the extent of rectal involvement and the ability to achieve a viable distal stump. A critical element to consider is the ability to safely expose pelvic anatomy. Important structures that require adequate visualization and protection include the musculature of the pelvic floor (to ensure continence), urological organs contiguous to the rectum (e.g., ureters, trigone, and urethra), and presacral vessels. Patient stability should be factored in when determining type of surgery and extent of resection. Other factors related to the patient include age, associated comorbidities, and history of risk factors of colon ischemia. These may place the patient in a high-risk group for postoperative complications as the extent of resection is an important indicator of postoperative course. We agree with Nelson et al. that Hartmann's procedure and low division of the rectum is the procedure of choice in an emergency setting if a viable rectal stump is feasible. Based on the limited number of cases seen in the literature, APR seems to be associated with more prohibitive mortality. This case report demonstrates that not resecting ischemic rectum in the context of hostile anatomy is a reasonable approach. The principle supporting this is that the remaining necrotic rectal tissue usually drains through the anus. The patient will likely not develop generalized peritonitis as the infection is contained within the pelvis. However, as illustrated in this case report, it is important to achieve the three following elements: (1) stool diversion through a proximal end colostomy, (2) Wide drainage of the affected quadrant, and (3) mucus fistula to allow sufficient residual drainage.
